# A Systematic Study of the Factors Affecting the Surface Quality of Chemically Vapor-Deposited Diamond during Chemical and Mechanical Polishing

**DOI:** 10.3390/mi15040459

**Published:** 2024-03-28

**Authors:** Zewei Yuan, Zhihui Cheng, Yusen Feng

**Affiliations:** School of Mechanical Engineering, Shenyang University of Technology, Shenyang 110870, China; zhihuicheng99@163.com (Z.C.); fys000421@163.com (Y.F.)

**Keywords:** surface quality, chemical mechanical polishing, diamond, mechanical polishing

## Abstract

Diamond surfaces must be of high quality for potential use in semiconductors, optical windows, and heat conductivity applications. However, due to the material’s exceptional hardness and chemical stability, it can be difficult to obtain a smooth surface on diamond. This study examines the parameters that can potentially influence the surface quality of chemically vapor-deposited (CVD) diamonds during the chemical and mechanical polishing (CMP) process. Analysis and experimental findings show that the surface quality of polished CVD diamonds is significantly influenced by the crystal structure and the growth quality of the diamond. In particular, when the surface roughness is below Ra 20 nm, the pores and grain boundaries on CVD diamond obstruct surface roughness reduction during mechanical polishing. To obtain a smooth polished surface, careful consideration of the size of diamond abrasives and polishing methods is also a prerequisite. Chemical mechanical polishing is a novel method to achieve a surface quality with roughness below Ra 3 nm, as in this method, the anisotropy of the CVD diamond allows the uneven steps to be efficiently erased. However, the chemical actions of polishing slurry should be controlled to prevent the formation of chemical etching pits.

## 1. Introduction

Diamond possesses exceptional physical, thermal, optical, and chemical properties, including excellent hardness, good chemical inertness, high thermal conductivity, a high elastic modulus, lager electrical resistance, a wide electronic gap, wide-range transparency, and a low friction coefficient [1]. It has widespread applications in electrical semiconductors, optical windows, high-fidelity loudspeakers, high-energy accelerators, and so on [2,3,4]. Diamond’s high heat conductivity of 2000 Wm^−1^ K^−1^ and bandgap of 5.5 eV make it an appropriate choice to serve as a semiconductor substrate [5]. Diamond has outstanding resistance to irradiation damage, significant thermal conductivity, and broad optical transmissivity from ultraviolet to far-infrared light, making it a promising material for high-power infrared lasers and X-ray detectors [6]. Diamond-based electronic equipment in spacecraft can withstand high-altitude ionizing radiation, thermal radiation, and cosmic rays due to its high radiation resistance [7]. Diamond is an effective material utilized in fusion facilities for its excellent capacity to transmit light and heat; diamond windows can readily pass through 2 MW of microwave energy [8,9]. Diamond’s high elastic modulus and low density allow it to transmit sound at the highest possible speed of 10,000 m/s. For high-frequency and high-power SAW devices, diamonds can therefore be used [10,11]. For ultraviolet and radiation detectors, diamonds’ wide bandgap, high melting point, strong breakdown field strength (10^7^ V/cm), and wide range transparency are advantageous [12,13]. In addition, due to their extreme hardness and exceptional chemical inertness, diamonds make suitable electrodes in chemical fields [14,15].

Regardless of the diamond’s outstanding features, if the surface quality is poor, the benefit of using the diamond’s distinctive characteristics will be nullified for particular industrial applications. To accomplish atomic connection, for instance, a semiconductor substrate needs a diamond with a flat surface and surface roughness below Ra 1 nm [16]. Large-sized diamond wafers can only be used for surface acoustic wave filters if their surface can be polished to a surface roughness of about Ra 1~3 nm with good flatness. Furthermore, as an optical window, the diamond should have a fine surface free of pits and mechanical scratches to limit the absorption of the infrared laser and X-rays [17]. Diamond surfaces with smooth roughness profiles are beneficial to extend the life of diamond cutters in the field of ultra-precision machining [18]. To reduce the frictional coefficient, the diamond’s wear and friction fields also require a flat surface. So, polishing and planarization without mechanical scratches and chemical etching pits are the crucial procedures to realize the preceding applications for the diamond [19].

To achieve a good-quality diamond surface, numerous polishing methods have been developed, including mechanical polishing (MP), thermochemical polishing (TCP), chemical mechanical polishing (CMP), dynamic friction polishing (DFP), laser polishing (LP), ion-beam polishing (IBP), and so on [20,21]. These methods can be split into five categories based on their material removal mechanisms: chemical reactions, evaporation, sputtering, micro-chipping, and the conversion of diamond into graphite [22]. The process of micro-chipping is the primary mechanism used in mechanical polishing and grinding to remove diamonds. Diamond abrasives easily scrape the surface, causing fracture pits and scratches [23]. Thermochemical polishing and dynamic friction polishing provide a higher rate of material removal to transform diamond into graphite through the catalytic action of transition metals such as iron, manganese (Mn), lanthanum (La), cerium (Ce), and its alloys [24,25]. Using this method, residual graphite can be easily retained on the diamond surface. Thin diamond films shatter under unsteady polishing-plate pressure. There are two non-contact polishing techniques: ion beam polishing and laser polishing. By using the diamond’s high-temperature evaporation, laser polishing eliminates the diamond [26,27]. A thin diamond film breaks due to a higher temperature gradient, and processed diamonds may have a layer of graphite on the surface. Physical ion sputtering is used in ion beam polishing to remove diamond atoms [28]. Many scanning waves remain on the surface of a diamond. Chemical mechanical polishing accelerates the material removal by adding a lot of chemical agents into the slurry, including NaNO_3_, KNO_3_, KOH, KClO_3_, K_2_Cr_2_O_7_, H_2_O_2_, HClO, HNO_3_, H_2_SO_4_, AgO, Cr_2_O_3_, MnO_2_, BaO_2_, PdO_2_, and their mixtures [29,30,31]. If too many chemical agents are introduced to the slurry, the intense chemical action causes the formation of a large number of chemical etching pits on the diamond surface. It can be deduced that surfaces treated with various techniques differ greatly from each other. The characteristics of different processes have a considerable impact on the quality of the processed surface. So, it is important to investigate the parameters that influence diamond surface quality to meet the requirements for various applications.

Mechanical polishing and chemical mechanical polishing are common low-cost and ultra-smooth methods to achieve the planarization of large-sized diamonds. The current work aims to provide a systematic analysis of the variables that affect diamond surface quality throughout chemical and mechanical polishing procedures, bringing the authors’ ten years of diamond polishing research to a conclusion. The mechanisms generating defects on diamond surfaces during these polishing procedures are examined, and a strategy is proposed to eliminate processing faults to obtain a smooth diamond surface. The results of this work apply to several domains of physical, thermal, optical, and electronic semiconductors to satisfy different surface quality criteria.

## 2. Materials and Methods

### 2.1. Diamond Sample Preparation

The diamond samples were produced using the chemical vapor deposition (CVD) process. To examine the effects of crystal structure on the quality of polished surfaces, experiments were conducted with two types of CVD diamond: single-crystal diamond and polycrystalline diamond. The single-crystal diamonds were 0.8 mm thick and then cut into sizes of 5 mm × 5 mm. The surface roughness of single-crystal diamond was reduced to about Ra 5 nm after mechanical lapping. The polycrystalline diamonds were produced by the DC Arc Plasma Jet method. Two-inch samples with a thickness of one millimeter were used for the polishing experiments. The average roughness of polycrystalline diamonds was up to Ra 15 µm or Rz 180 µm. So, it was necessary to remove a layer of at least 180 µm to obtain a smooth surface.

### 2.2. Methods of Mechanical Polishing and Grinding

Mechanical polishing and grinding are effective methods to remove rough asperities from polycrystalline diamonds. In the mechanical polishing experiment, rough asperities on the diamond surface were removed by performing a series of polishing stages using the corresponding W10, W5, W2, W0.5, and W0.1 diamond powders. The mechanical polishing investigations were carried out using diamond lapping and polishing equipment developed by Shenyang University and Technology. A rotational speed of 750 r/min was used for the stage of lapping with diamond abrasives. Ceramic-based diamond grinding wheels of 2000# and 10,000# were used for the grinding of diamond. The rotational speed of grinding was about 5000 r/min.

### 2.3. Method of Chemical Mechanical Polishing

Chemical mechanical polishing was used to fine polish CVD diamonds. The slurry for chemical mechanical polishing is primarily composed of oxidant, abrasive, additive, and deionized water. The oxidability of common oxidants can be represented by standard electrode potentials; the higher the standard electrode potential, the greater the oxidability. The oxidation fading test of methyl orange can also characterize the oxidizability of the slurry. If the slurry has strong oxidizability, the color of the methyl orange solution will fade out. According to previous studies [16,19], 30 g of K_2_FeO_4_ and 10 mL of H_2_O_2_ in 500 mL of deionized water were used as the oxidant for the stage of CMP. Phosphoric acid and ferrous sulfate solutions were added into the slurry to accelerate the chemical reaction. Boron carbide powder with a particle size of 0.2 μm was employed as a CMP abrasive. In order to avoid the decomposition of K_2_FeO_4_ and H_2_O_2_, a solution of phosphoric acid and ferrous sulfate was dropped on the surface of polishing plate.

### 2.4. Characterizations of Surface Quality

The morphology of the diamond surface before and after polishing was analyzed using an OLUMPUS laser scanning confocal microscope manufactured in Tokyo, Japan. The surface roughness of the diamond was measured using the Mitutoyo SJ-411 surface topography profiler made by Mitutoyo (Kawasaki, Japan) and the Zygo New View 5022 surface topography profiler made by Zygo (Middlefield, CT, USA).

## 3. Results and Discussion

### 3.1. Influence of CVD Diamond Grain Growth

**Figure 1:**
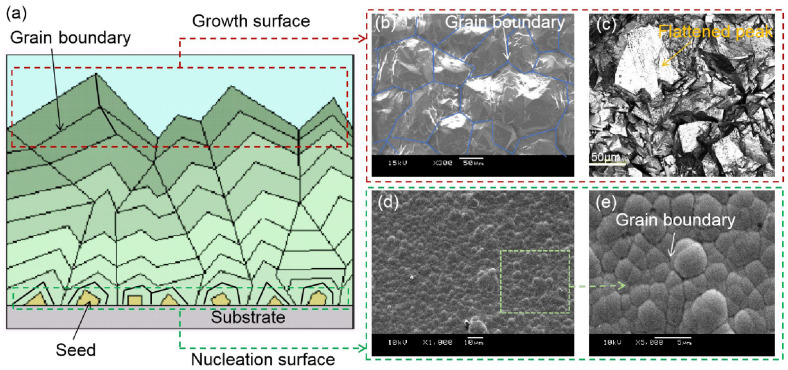
Microstructure of CVD diamond on growth surface and nucleation surface (**a**) the growth schematic diagram of CVD diamond; (**b**,**c**) the morphology of the growth surface; (**d**,**e**) the morphology of nucleation surfaces.

CVD diamond exhibits a relatively rough surface due to the limitations of grain growth. The surface roughness of CVD diamonds is affected by growth time and speed; it usually increases with growth time and speed. Figure 1 depicts a schematic diagram of the growth mechanism, as well as the morphology of the growth and nucleation surfaces. As seen in the figure, the surface roughness on the growth surface is often more than that on the nucleation surface due to the larger crystal grain. The surface roughness and asperity height can reach Ra 13~18 μm and 180 μm, respectively. All the original asperities must be removed throughout the polishing process to create a smooth surface. So, the removal height of polishing is usually determined by the highest asperity and waviness of the diamond sample. When the diamond surface is quite rough, it will take a long time to obtain an ideal surface using the mechanical polishing or CMP processes. In addition, the surface of a CVD diamond has a large number of grain boundaries, as shown in Figure 1b–e. The grain boundaries make it very difficult to reduce surface roughness during the fine-polishing stage. Furthermore, the disparity in the grain’s growth has a detrimental effect on the reduction of surface roughness during mechanical polishing. Grain boundaries are also the most important elements influencing the surface quality of CVD diamonds on the nucleation surface. To create a smooth surface, all nucleation layers must be removed.

### 3.2. Influences of Different Polishing Methods

**Figure 2:**
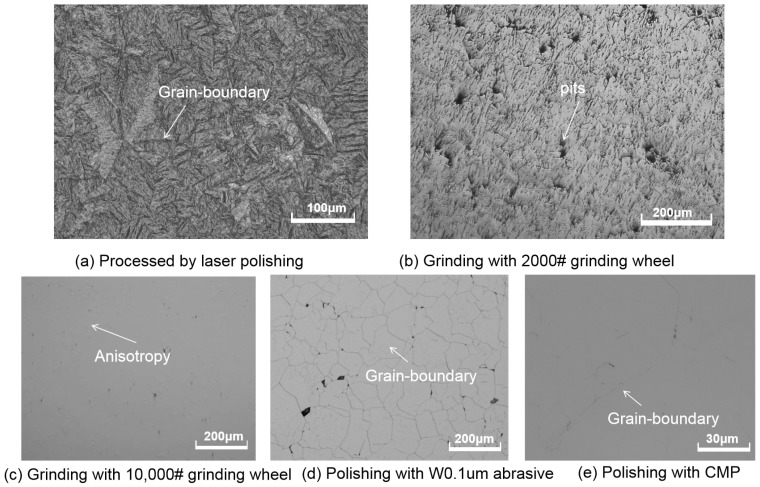
Morphology of CVD diamonds processed with different mechanical and CMP methods.

The surface morphology of different polishing processes varies based on different mechanisms. Figure 2 depicts the morphology of CVD diamonds produced using grinding, mechanical polishing, and CMP methods. Figure 2a shows that all initial asperities were removed after twenty minutes of laser polishing but the surface grain boundaries were still visible. The surface roughness of CVD diamonds produced with laser polishing ranged from 200 to 600 nm. A 2000# grinding wheel was then used to process the CVD diamonds. The surface brightness was increased compared to laser polishing, and the roughness was reduced to approximately Ra 120~200 nm. As seen in Figure 2b, the diamond surface contained numerous pits and scratches. When the diamond sample was continuously treated with a 10,000# diamond grinding wheel, the surface became smoother, as seen in Figure 2c. There were no visible scratches on the surface, and the roughness decreased to Ra 10 nm. If the grinding wheel becomes dull, a large number of elevated regions will appear on the surface. As a result, it is essential to maintain the grinding wheel’s sharpness during the process. Figure 2d depicts the nucleation surface morphology of a diamond polished with W0.1 diamond abrasive. On the surface, there are several grain boundaries, and the surface roughness is reduced to around Ra 6~8 nm. Chemical mechanical polishing is a popular fine-polishing process for most semiconductor materials, as it uses the synergistic effects of both chemical and mechanical processes to eliminate diamond atoms. Figure 2e shows that the surface of the diamond formed using the CMP process was very smooth, with a roughness of about Ra 3~5 nm with evident grain boundaries on the surface.

### 3.3. Influence of Diamond Abrasive Size

**Figure 3:**
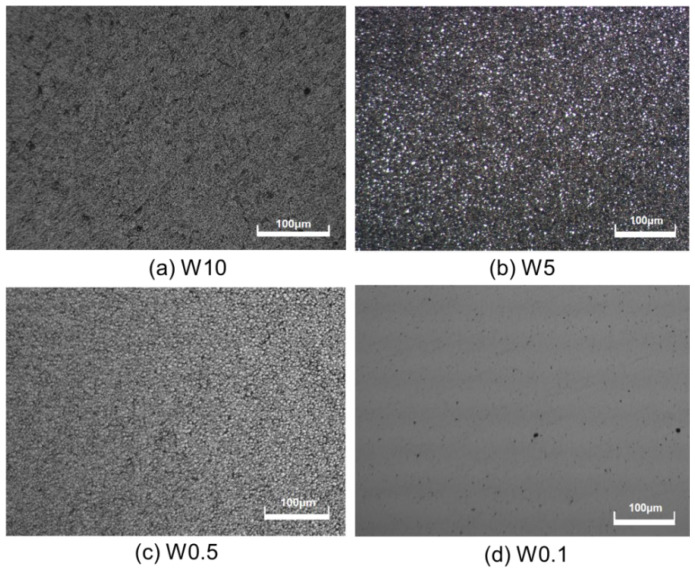
Morphologies of CVD diamonds polished with different diamond abrasives.

**Figure 4:**
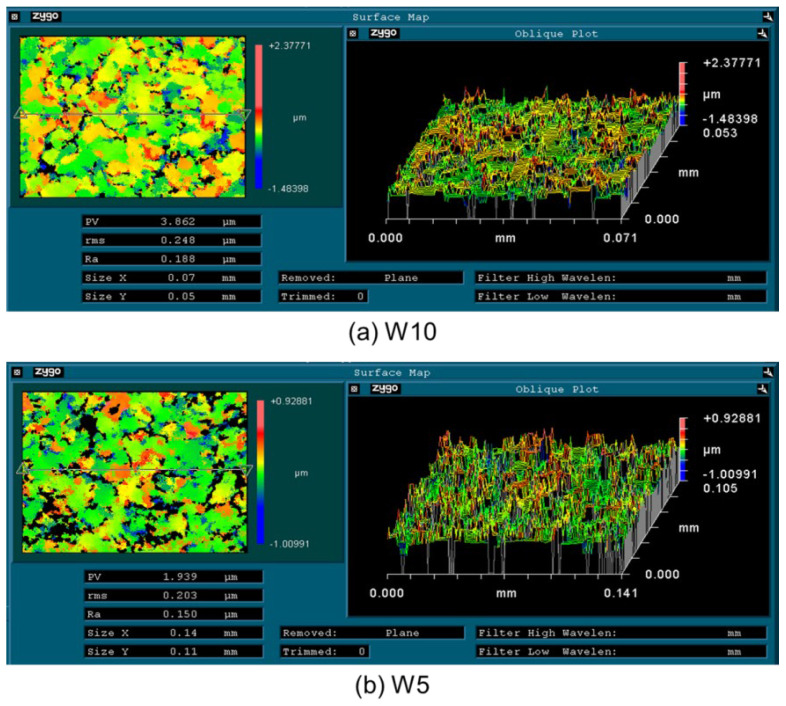
Surface maps of CVD diamonds polished with W10 and W5 diamond abrasives.

**Figure 5:**
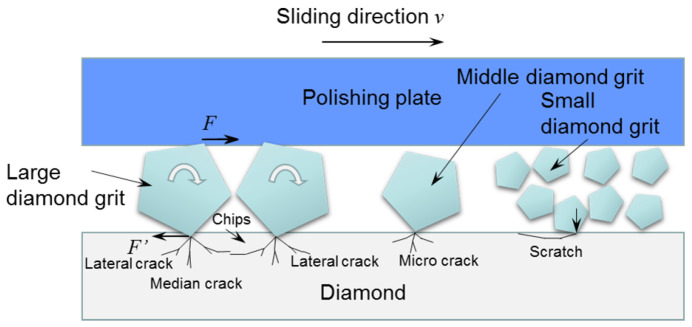
Schematic diagram of contact state with W10 and W5 diamond abrasives.

The size of the diamond’s abrasive has a considerable impact on its surface quality and the material removal rate during mechanical polishing. The material removal rate increases with the size of the diamond abrasive. On the other hand, as the abrasive size decreases, the surface roughness significantly reduces. Figure 3 displays the morphology of a polished CVD diamond using various diamond abrasives. After polishing with W10 and W5 abrasives, all original asperities on the diamond surface were removed. When diamond samples were polished with a W0.5 abrasive, raised micro-regions became visible. Extensive polishing with W0.1 abrasive reduced diamond surface roughness to Ra 8 nm. When polished with W10 and W5 abrasives, it was found that the diamond surface had many pits; pits could be readily examined with the New View 5022 surface morphology profiler. Figure 4 shows that the diamond surface had several fracture pits but no mechanical scratches after being polished with W10 and W5 abrasives. This implies that mechanical polishing with W10 and W5 abrasives uses three-body contact modes. Diamond fractures by abrasive indentation and the depths of fracture pits created by W10 and W5 abrasives were around 1.0 and 0.5 μm, respectively. When a diamond is lapped with W0.5 and W0.1 abrasives, the diamond grits cannot be in contact with the diamond sample and the polishing plate simultaneously. As demonstrated in Figure 5, small diamond grits scratch and support one another on the diamond surface. Small diamond grits exert less force on diamonds than large diamond grits, thus making the fracturing of diamonds difficult. As a result, the diamond sample presents a smooth surface as shown in Figure 3d.

### 3.4. Influence of Polishing Time

**Figure 6:**
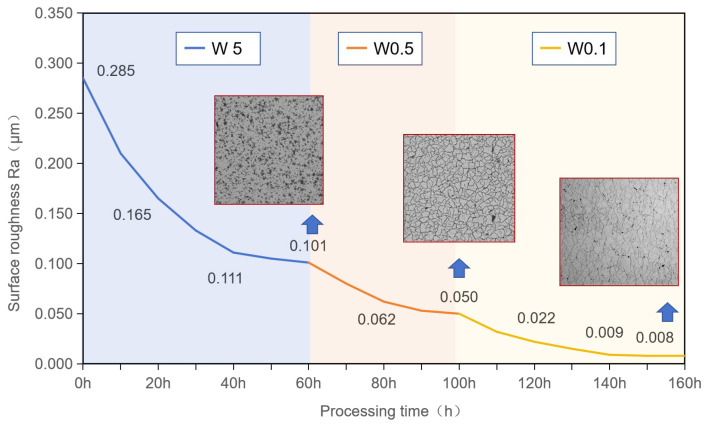
Surface roughness of CVD diamond varying with polishing times.

Due to diamond’s extreme hardness and chemical inertness, diamond polishing takes a long time to achieve the desired surface roughness. So, establishing a proper link between different-sized abrasives is vital. Figure 6 shows how the surface roughness of CVD diamond varies with mechanical polishing time. Within the first thirty hours of polishing using W5 abrasives, the surface roughness significantly decreased with polishing time. After polishing for 30 h again, the curve remained steady in terms of surface roughness reduction. It was difficult for W5 abrasives to further reduce surface roughness below Ra 0.1 μm. Similarly, W0.5 and W0.1 abrasives had the same trends as W5 abrasives. The limits of surface roughness polishing with W0.5 and W0.1 were about Ra 50 nm and Ra 8 nm, respectively. It was difficult to further reduce surface roughness since polishing took a long time, with no significant reduction in surface roughness. Proceeding from the discussion, it can be inferred that linking the polishing time of various abrasives is important.

**Figure 7:**
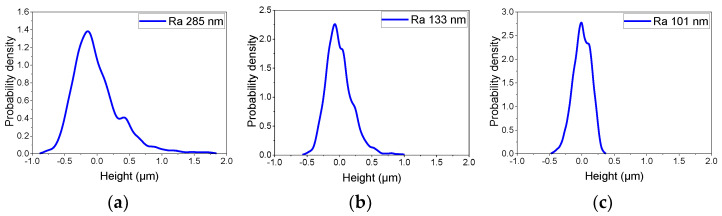

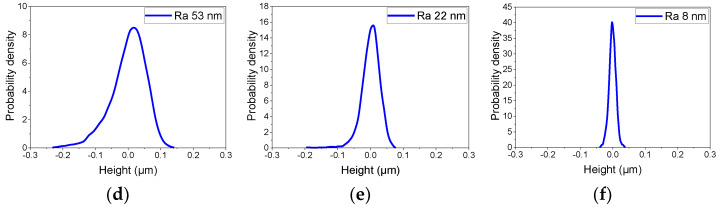
Asperity height distributions of CVD diamonds processed with different abrasives and time durations. (**a**) Grinding; (**b**) polishing for 30 h with W5; (**c**) polishing for 60 h with W5; (**d**) polishing for 90 h with W5 and W0.5; (**e**) polishing for 120 h with W5 and W0.5; (**f**) polishing for 150 h with W5, W0.5, and W0.1.

The asperity height distribution of CVD diamond, as shown in Figure 7, indicates that different abrasives have a limit in their ability to reduce surface roughness. The surface asperity height distributions differed when CVD diamond samples were processed with various abrasives. Due to the presence of grinding grooves, the surface asperity height of the ground surface had a skewed normal distribution. When the diamond was polished using W5 abrasive, a normal distribution with a surface roughness of Ra 101 nm reflected the height distribution of the CVD diamond’s surface asperity. When W5 abrasives were replaced with W0.5 and W0.1 abrasives, a skewed normal distribution formed, which eventually transitioned to normal distribution with a surface roughness of Ra 8 nm after adequate polishing. So, the surface asperity height distributions reflect the unique stable polishing conditions.

### 3.5. Influence of Crystal Anisotropy

**Figure 8:**
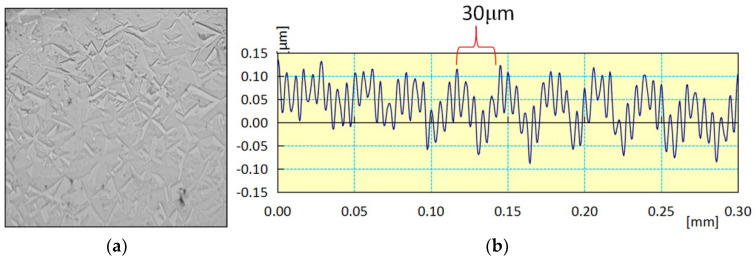

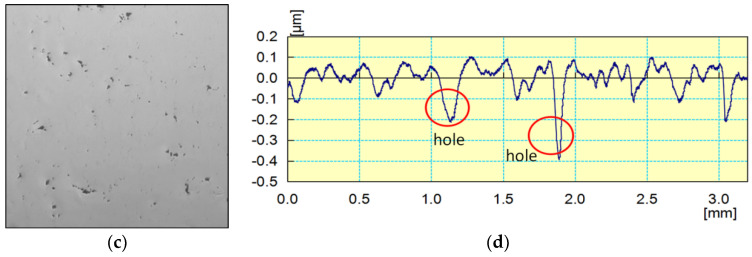
Morphologies and roughness profiles of CVD diamonds processed with MP and CMP. (**a**) Morphology of MP; (**b**) roughness profile of MP; (**c**) morphology of CMP; (**d**) roughness profile of CMP.

Diamond has high anisotropy, and its hardness on different crystal planes ranges from 80 to 120 GPa. As such, the material removal rates vary when mechanical polishing is performed on different crystal surfaces [32]. Diamond exhibits polycrystalline and anisotropy during chemical vapor deposition if the process parameters are not monitored closely. As a result, irregular protrusions were distributed across the surface of the CVD diamond after polishing with MP, as shown in Figure 8a. The height of these protrusions was approximately 0.1 μm, as seen in Figure 8b. Apart from diamond anisotropy, the height of these protrusions was closely related to the hardness of the grinding wheel or polishing plate. When CVD diamond is polished using a hard polishing plate, the protrusion on the surface becomes difficult to observe. However, these protrusions can be removed using the CMP process. Figure 8c shows no obvious protrusions on the surface. This indicates that the anisotropy of CVD diamond does not affect the CMP process, provided that the CMP parameters are reasonably controlled. Otherwise, etching pits will form on the polishing surface if the chemical reaction is extremely powerful, as seen in Figure 8d. Etching pits are typically generated by holes at grain boundaries. So, the preparation of appropriate CMP slurry is required for various CVD diamond samples. The chemical action cannot be too strong for CVD diamonds with a large number of original holes. If the growth quality of the CVD diamond is good, the chemical action can be enhanced to increase the material removal rate.

### 3.6. Influence of Diamond Grain Boundaries and Pores

**Figure 9:**
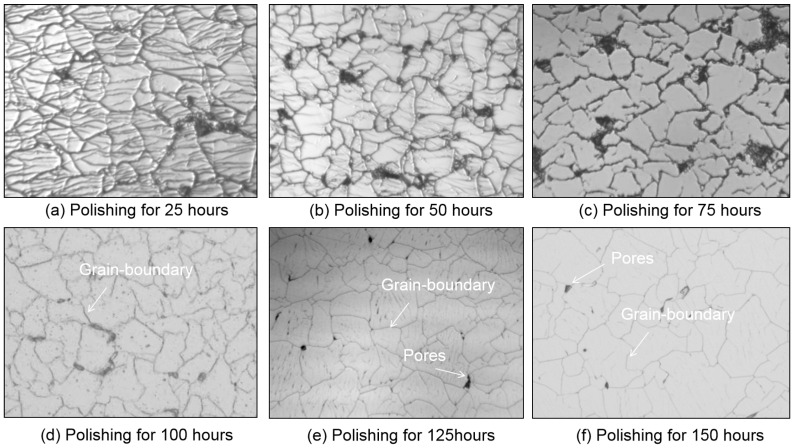
Morphology of CVD diamond polished for different times.

A large number of grain boundaries are formed during the formation of polycrystalline CVD diamonds. When the surface roughness is reduced to particular values, these grain boundaries greatly influence the surface quality of CVD diamonds. Figure 9 shows the morphology of a CVD diamond polished for different time durations with W0.1 abrasive. Diamond samples for this stage were polished using W5 and W0.5 abrasives. As shown in the image, many reticulated cracks appeared on the surface of the diamond after polishing with W0.1 abrasive. These reticulated cracks represent the CVD diamond’s polycrystalline structure. Polishing refines the grain boundaries and during the procedure, the diamond’s surface roughness also decreases. Figure 9a,b show some unidirectional cracks on the grain surface of the diamond; these unidirectional cracks were caused by mechanical polishing with large abrasives and could be removed with fine abrasives. Furthermore, certain pores could be observed when grain boundaries overlapped during the diamond growth process and eventually caused an increase in surface roughness during the fine-polishing process.

### 3.7. Influence of Single-Crystal and Polycrystalline Diamonds

**Figure 10:**
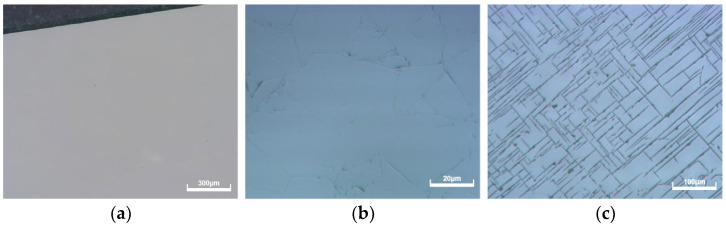
Morphologies of CVD diamonds polished for different times. (**a**) Single-crystal diamond; (**b**) polycrystalline diamond; (**c**) single-crystal diamond with scratches.

Diamond surface quality is significantly influenced by its crystal structure. When compared to polycrystalline diamond, single-crystal diamond makes it easier to create a very smooth surface, as demonstrated in Figure 10a, because the polishing process is independent of crystal anisotropy, grain boundaries, and porosity, and surface roughness can be decreased to less than Ra 1 nm. Preparing large single-crystal diamonds is difficult because of their higher cost and longer production time than polycrystalline diamonds. As illustrated in Figure 10b, the crystal anisotropy and grain boundaries of polycrystalline diamonds are the primary factors influencing surface roughness. Furthermore, when the polycrystalline diamonds were polished using the mechanical polishing method, the surfaces at the edge of the diamond samples showed many scratches. Unlike polycrystalline diamonds, the scratches had an interlaced distribution on the diamond surface, as seen in Figure 10c. Scratches are mostly caused by large diamond abrasives; when a diamond is polished with a diamond abrasive with a size less than 0.1 μm, there are no scratches on the diamond surface. The distribution of these scratches is closely related to the diamond’s crystal orientation, as the hardness of crystals varies with their orientation. Therefore, for mechanical polishing, the influence of crystal orientation must be considered.

### 3.8. Influence of Polishing Parameters

**Figure 11:**
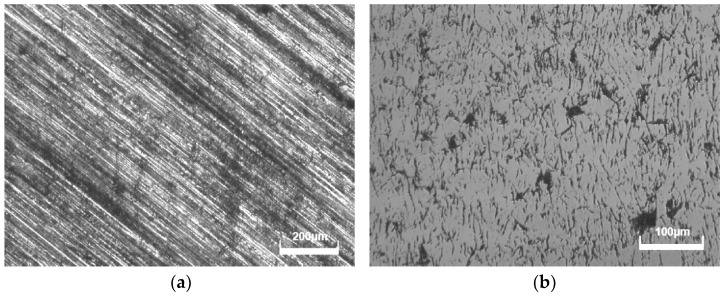
Morphologies of CVD diamonds polished with and without sample rotation. (**a**) Without sample rotation; (**b**) with sample rotation.

The primary parameters of mechanical polishing are the rotational speed of the polishing plate and sample, polishing pressure, and polishing time. Chemical mechanical polishing is more complex than mechanical polishing, as it includes quantities of polishing slurry and chemical reagents. Shifting the polishing pressure and rotating speed during the mechanical polishing process significantly improves diamond material removal. However, excessive polishing pressure can cause cracks in diamonds. To achieve a considerable rate of material removal, increasing the rotational speed of the polishing plate is suggested. Furthermore, the rotation of the diamond sample is critical for maintaining optimal surface quality. Figure 11 depicts the morphology of CVD diamonds polished with and without sample rotations. When the sample was not rotated during mechanical polishing, it produced a large number of unidirectional scratches on the surface. When samples were rotated during polishing, the number of scratches fell dramatically, and the surface roughness decreased compared to non-rotated samples. To achieve a high-quality large-sized diamond wafer, the polishing process should be carried out with the diamond sample’s rotation. It should be noted that the rotation accuracy of the polishing plate must be carefully managed to ensure that the entire diamond surface is polished.

**Figure 12:**
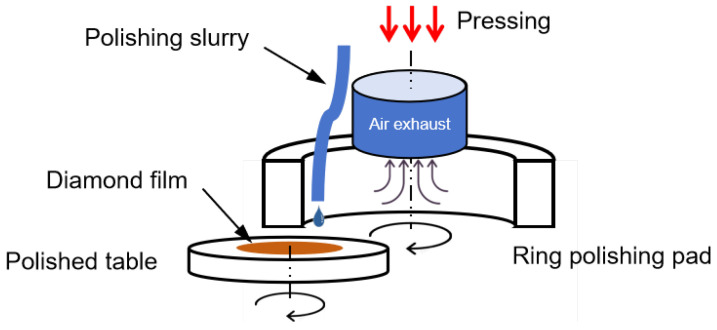
Schematic diagram of CMP polishing.

**Figure 13:**
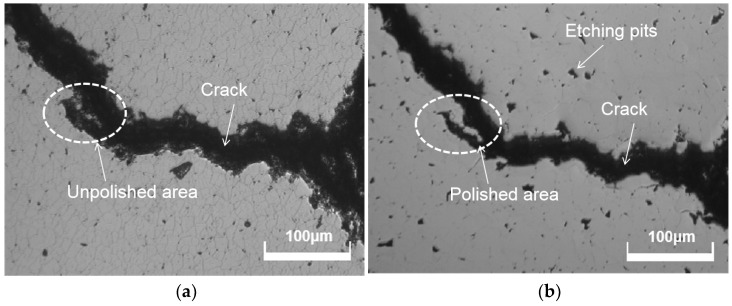
Morphologies of CVD diamonds polished with mechanical polishing and CMP. (**a**) Mechanical polishing; (**b**) chemical mechanical polishing.

During the chemical mechanical polishing process, the chemical action influences not only the rate of material removal but also the surface quality of the diamond. A slurry with strong chemical action can speed up the material removal process. However, some etching holes will form on the diamond’s surface as a result of the excessive chemical action. As shown in Figure 12, when phosphoric acid and ferrous sulfate solutions were added into the slurry, Fenton’s reagent was generated on the diamond surface. Fenton’s reagent has very strong oxidizability. It can etch the diamond, especially the material at the edges of grain boundaries and pores. Figure 13 depicts a fracture on the diamond surface to compare the morphologies of CVD diamonds polished with mechanical polishing and chemical mechanical polishing. According to Figure 13a, many grain boundaries were detected on the diamond surface after mechanical polishing. When the diamond sample was polished with a strong chemical slurry, the grain boundaries disappeared, but the strong chemical activity left several chemical etching pits on the diamond surface. To achieve a good surface quality, it is necessary to manage both chemical and mechanical action during the CMP process.

## 4. Conclusions

Mechanical and chemical mechanical polishing (CMP) methods are commonly used to smooth large-size diamonds in semiconductors, optical windows, and heat conductivity; nonetheless, generating a smooth diamond is quite challenging for several reasons. The present study systematically investigated the parameters affecting the surface quality of chemically vapor-deposited (CVD) diamonds during the chemical and mechanical polishing process based on prior work on diamond polishing. It aims to provide useful recommendations for the applications of large-sized diamonds in semiconductors, optical windows, and heat conductivity. The conclusions are summarized below:

(1) The crystal growth structure of CVD diamond not only determines its original surface roughness but also has a substantial impact on polishing surface quality. Polycrystalline diamonds’ crystal anisotropy leads to certain steps on the CVD diamond surface due to the varying removal rates in different crystal planes. These steps increase the surface roughness of CVD diamonds during mechanical polishing. Chemical mechanical polishing is effective in removing these steps.
(2) Grain boundaries and holes appear on polycrystalline diamonds when the surface roughness is less than Ra 20 nm. These grain boundaries and holes are the primary variables responsible for increasing CVD diamond surface roughness values during mechanical polishing.
(3) Chemical mechanical polishing is a unique technique to achieve surface quality with a roughness of less than Ra 3 nm, as the anisotropy of the CVD diamond efficiently erases the uneven steps. However, the chemical actions of polishing slurry need to be controlled to prevent the formation of chemical etching pits.
(4) As the anisotropy, grain boundaries, and pores do not interfere with the polishing process, single-crystal diamonds with Ra values less than 3 nm are readily polished.
(5) To obtain a smooth polishing surface, rough and fine-polishing techniques must be carefully planned. A suitable connection should be made between the polishing phases for variable abrasive sizes.

## Data Availability

Data is contained within the article.
